# Supplementary material for: Resolving the full spectrum of human genome variation using Linked-Reads
Source: Genome Res. 2019 Apr;29(4):635–45. doi: 10.1101/gr.234443.118 (PMC6442396; doi:10.1101/gr.234443.118)
Supplement: Supplemental Material [file supp_gr.234443.118_Supplemental_longranger-2.2.zip › longranger-2.2.tar.bz2!/lr2.2-public/lib/stan/cmdstan/stan/doc/api/html/expression07__grammar_8hpp.html]

Stan: src/stan/gm/grammars/expression07\_grammar.hpp File Reference


|  |  |
| --- | --- |
|  | Stan  2.5.0  probability, sampling & optimization |


- Main Page
- Related Pages
- Namespaces
- Classes
- Files

- File List
- File Members

All Classes Namespaces Files Functions Variables Typedefs Enumerations Enumerator Friends Macros Pages

- src
- stan
- gm
- grammars

Classes |
Namespaces

expression07\_grammar.hpp File Reference

`#include <string>`  
`#include <sstream>`  
`#include <vector>`  
`#include <boost/spirit/include/qi.hpp>`  
`#include <stan/gm/ast.hpp>`  
`#include <stan/gm/grammars/expression_grammar.hpp>`  
`#include <stan/gm/grammars/term_grammar.hpp>`  
`#include <stan/gm/grammars/whitespace_grammar.hpp>`

Go to the source code of this file.

|  |  |
| --- | --- |
| Classes | |
| struct | stan::gm::term\_grammar< Iterator > |
|  | |
| struct | stan::gm::expression\_grammar< Iterator > |
|  | |
| struct | stan::gm::expression07\_grammar< Iterator > |
|  | |

|  |  |
| --- | --- |
| Namespaces | |
|  | stan |
|  | Probability, optimization and sampling library. |
|  | |
|  | stan::gm |
|  | Directed graphical model compiler. |
|  | |


---

[ Stan Home Page ]

*© 2011–2014,
Stan Development Team.*
